# Identification and validation of a potential key gene SGOL1 for poor prognosis in hepatocellular carcinoma based on a bioinformatics approach

**DOI:** 10.3389/fonc.2022.1043161

**Published:** 2022-11-09

**Authors:** Xiaobin Fei, Songbai Liu, Peng Liu, Xing Wang, Changhao Zhu, Junyi Hou, Junzhe Cai, Yaozhen Pan

**Affiliations:** ^1^ School of Clinical Medicine, Guizhou Medical University, Guiyang, Guizhou, China; ^2^ Department of Hepatobiliary Surgery, Guizhou Cancer Hospital, Guiyang, China

**Keywords:** SGOL1, hepatocellular carcinoma, bioinformatics analysis, immune cell infiltration, poor prognosis

## Abstract

**Objective:**

Hepatocellular carcinoma (HCC) is one of the most prevalent types of cancer worldwide. Shugoshin 1 (SGOL1) plays a crucial role in cell mitosis and its aberrant expression level in human tumors has shown to promote chromosomal instability (CIN) and accelerate tumor growth. SGOL1 expression level in HCC cells and tissues, whether it has an influence on HCC patients’ prognosis, and its mechanism of action have not yet been studied.

**Methods:**

We carried out the bioinformatics analysis of SGOL1 expression level and survival analysis in 8 different malignancies, including HCC. In addition, we analyzed SGOL1 expression level in HCC tissues, as well as HCC patients’ clinical features, enrichment analysis of SGOL1 function and mechanism of action in HCC and tumor immune cells. The effects of SGOL1 expression level and cell viability on HCC were confirmed by *in vitro* cytological assays.

**Results:**

It was found that SGOL1 mRNA expression level was significantly higher in several tumor tissues, including HCC, than in corresponding normal tissues, and the elevated SGOL1 expression level was strongly associated with HCC patients’ poor prognosis. It was also revealed that SGOL1 expression level in HCC tissue was positively correlated with disease stage, tumor grade, and tumor size, and the results of multivariate logistic regression analysis showed that SGOL1 was one of the independent influential factors of the prognosis of HCC. Enrichment analysis revealed that SGOL1 expression level in HCC tissue was mainly associated with tumor proliferation, cell cycle, and other factors. The results of the immune infiltration analysis indicated that SGOL1 expression level was associated with immune cell infiltration and immune checkpoints in HCC. *In vitro* experiments demonstrated the high SGOL1 expression level in HCC tissues and cells, and silencing of SGOL1 resulted in altered cell cycle markers and decreased proliferation, invasion, and migration of HCC cells.

**Conclusion:**

The findings revealed that SGOL1 is highly expressed in HCC tissues, it is a biomarker of a poor prognosis, which may be related to immune cell infiltration in HCC, and may enhance the proliferation, invasion, and migration of HCC cells. The results may provide new insights into targeted treatment of HCC and improve HCC patients’ prognosis.

## Introduction

**Figure 1:**
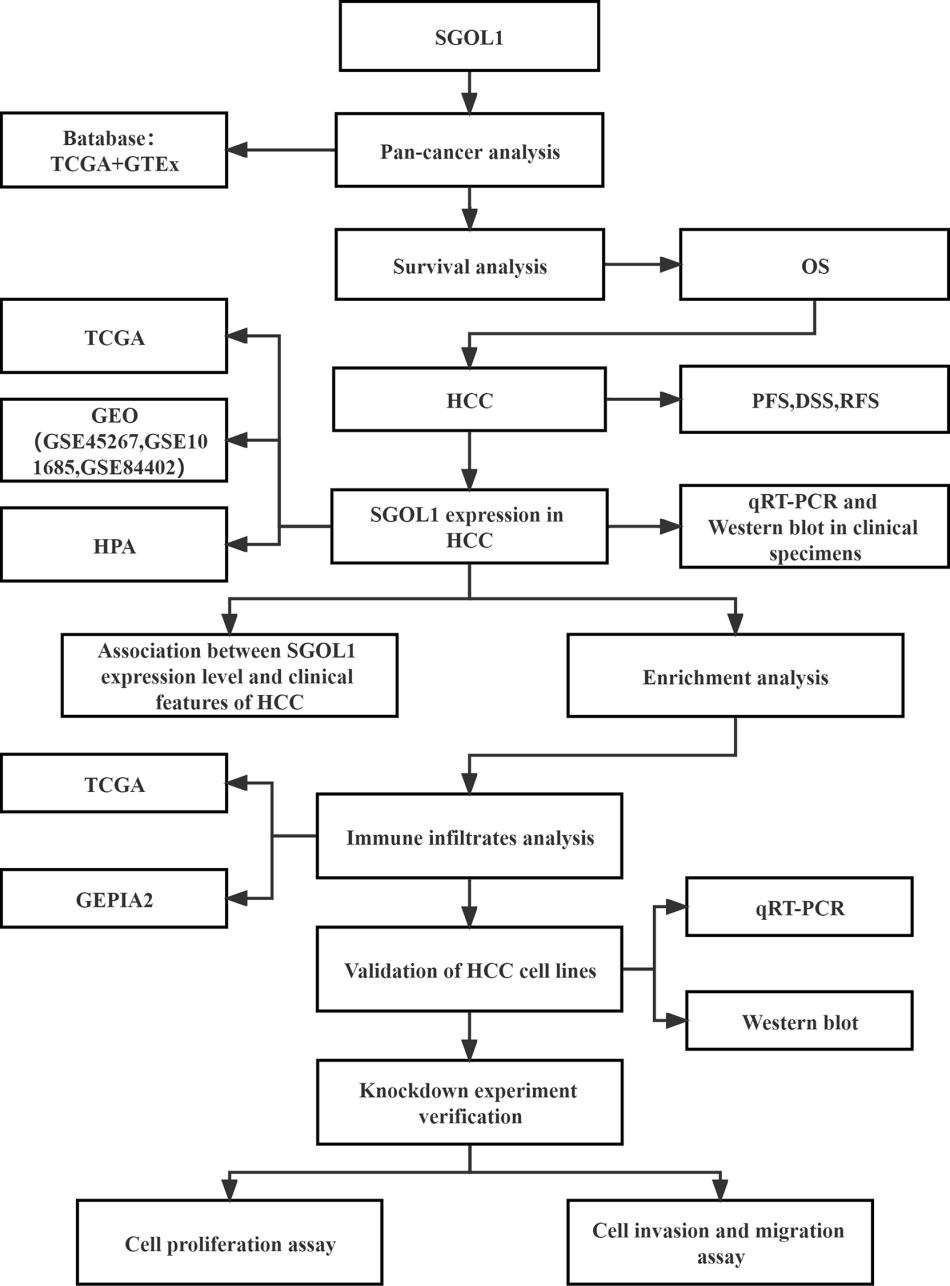
Workflow diagram of the data sources and methodological applications used in this study.

With identification of more than 30,000 fatalities and 40,000 new cases of primary liver cancer worldwide in 2022, hepatocellular carcinoma (HCC) is known as a global clinical challenge (1). It ranks third in incidence and fourth in mortality among malignant tumors of the digestive system (2). With the development of medical treatments in recent decades, liver cancer is treated by surgical resection, radiofrequency ablation, transcatheter arterial chemoembolization (TACE), liver transplantation, chemotherapy, and immunotherapy (3, 4). Despite the great progress of medical technologies in recent years, liver cancer has still a terrible prognosis, and overall survival rates of liver cancer patients have not been remarkably improved (5). HCC is the most common type of primary liver cancer (6). The primary risk factors for HCC included hepatitis B virus (HBV) infection, hepatitis C virus (HCV) infection, alcoholism, obesity, non-alcoholic liver disease, cirrhosis, and aflatoxin (7, 8), which has no specific symptoms in its early stages, as well as accompanying by a high recurrence rate and intra- and extra-hepatic metastases (9), and it is mainly detected at advanced stages. HCC has a wide range of pathophysiological characteristics, including abnormalities in cell cycle, DNA methylation, chromosomal instability (CIN), immunomodulation, epithelial-mesenchymal transition, an increase in the number of HCC stem cells, dysregulation of microRNAs (miRNAs), etc. (10, 11). Shugoshin 1 (SGOL1), a protein required for chromosomal segregation, frequently plays a substantial role in mitosis and meiosis (12), and is valuable for maintaining chromosome cohesion and preventing premature chromosome segregation and CIN (13). It has been found to play a role in the development of a number of malignancies, including its high expression level in progression of prostate cancer cells, promoting the development and metastasis of diseases through AKT-mediated signaling pathways (12). SGOL1 also plays a role in controlling multi-drug resistance in gastric cancer cells (14). In recent years, studies have profoundly concentrated on immune checkpoint inhibitors (ICIs), tumor immune microenvironment (TIME), and immune escape mechanisms (15). It was reported that the immunological microenvironment is crucial in the HCC progression. At present, ICIs are used in clinical settings, and they could remarkably improve the prognosis of HCC patients (16), thus, finding new tumor immunological targets is vital. Yamada HY et al. (13) showed a propensity for mild spontaneous lung and liver cancers by establishing a SGOL1(-/+)) mouse model of HCC with a dysregulated immune system. The mechanisms of tumor immunity are complex (17), and SGOL1 has rarely been studied in HCC and tumor immunity. In the present study, bioinformatics analysis was used to investigate SGOL1 expression level, prognosis, putative function, and its association with immune infiltration in HCC. The findings were verified using *in vitro* cellular assays, and it was found that SGOL1 promoted the development of HCC. In addition to its potent association with immune cells that infiltrate tumors, ICIs, and immune-infiltrating cell-related biomarkers for HCC, SGOL1 overexpression also plays a role in the invasion and metastasis of HCC cells *in vitro*. The study flowchart is shown in **Figure 1**.

## Materials and methods

### Gene expression datasets

Totally, 375 HCC and 39 normal liver tissue samples and matched clinical data were retrieved and downloaded from The Cancer Genome Atlas (TCGA) database (https://portal.gdc.cancer.gov/). GSE45267, GSE101685, and GSE84402, three microarray expression datasets, were obtained from the Gene Expression Omnibus (GEO) database (https://www.ncbi.nlm.nih.gov/geo/). These datasets were used to identify differences in SGOL1 expression level in HCC and normal tissues. In addition, RNA-seq data (level 3) for 8 cancerous and normal tissues were acquired from TCGA and Genotype-Tissue Expression (GTEx) (https://gtexportal.org/) databases for the pan-cancer study.

### Comprehensive analysis

#### Pan-cancer analysis

The following tumor types were selected for the pan-cancer analysis: invasive breast cancer (BRCA), colon cancer (COAD), esophageal cancer (ESCA), renal clear cell carcinoma (KIRC), hepatocellular liver cancer (LIHC), pancreatic cancer (PAAD), prostate cancer (PRAD), and gastric cancer (STAD). To analyze the correlation between SGOL1 mRNA expression level and prognosis in these tumors, differential expression analysis and survival analysis were performed using R 4.2.1 software. Specifically, differential expression analysis was carried out by “limma” and “edgeR” R packages, and survival analysis was conducted by “survival” and “survminer” R packages.

#### Immunohistochemistry

The immunohistochemical results of SGOL1 in HCC tissues were validated using the Human Protein Atlas (HPA) database (https://www.proteinatlas.org), providing immunohistochemical images of over 15,000 genes freely. To understand the differences in SGOL1 protein expression level between HCC and normal liver tissues, immunohistochemical images of the same antibody staining were extracted from this database.

#### Functional enrichment analysis

The David database (https://david.ncifcrf.gov/) was used to elucidate the potential gene ontology (GO) functions associated with SGOL1 expression level, including biological processes (BPs), molecular functions (MFs), and cellular components (CCs), as well as HCC-related pathway analysis using GESA-4. 1.0. False discovery rate (FDR)<0.05 was considered as the threshold. The association of SGOL1 expression level with tumor-related pathways was compared using the “GSVA” R package *via* single-sample gene set enrichment analysis (ssGSEA) and Spearman correlation analysis.

#### Immunoassay

The “immunedeconv” R package, a providing a reliable immune scoring assessment, was utilized to assess the correlation between SGOL1 gene expression level and 6 tumor-infiltrating immune cells (CD4^+^ T cells, neutrophils, macrophages, CD8^+^ T cells, B cells, and bone marrow-derived dendritic cells) in 375 HCC patients using the TIMER web resource. Using the gene expression profiling interactive analysis 2 (GEPIA2, http://gepia2.cancer-pku.cn/), a web-based program providing fast access to TCGA and GTEx databases (18), the association between the aforementioned cellular markers and SGOL1 expression level in HCC cells was assessed. In the meantime, the expression levels of immune checkpoint-related genes (SIGLEC15, TIGIT, CD274, HAVCR2, PDCD1, CTLA4, LAG3, and PDCD1LG2) in HCC cells were retrieved to determine the relationship between SGOL1 expression level and immune checkpoint-related genes. The above-mentioned results were visualized by “ggplot2” and “heatmap” R packages. The relationship between SGOL1 expression level and the transcript levels of the above-mentioned immune checkpoint-related genes was further validated by the GEPIA2.

#### Cell culture and transfection

From the Chinese Academy of Sciences (Beijing, China), the HCC cell lines (HUH-7, HepG2, Sk-Hep1, SMMC7721, and MHCC-97H) and the normal liver cell line (L02) were obtained. The cells were cultivated in a Dulbecco’s modified Eagle’s medium (DMEM) (Gibco, New York, NY, USA) enriched with 10% fetal bovine serum (FBS; BioInd, Israel), 1% penicillin, and streptomycin. They were grown in an incubator containing 5% CO_2_ at 37 °C. The target sequence of SGOL1siRNA-NC was (sense5’-UUCUCCGAACGUGUCACGUTT-3’; antisense5 ‘-ACGUGACACGUUCGGAGAATT-3’), the target sequence of SGOL1siRNA-1# was (sense5’-CCGCAAAUUCCUCUUGAAGAATT-3’;antisense5’UUCUUCAAGAGGAAUUUGCGGTT-3’), the target sequence of SGOL1siRNA-2# was (sense5’-AUAGCUGCACCAUGCCAAAUATT-3’;antisense5’-UAUUUGGCAUGGUGCAGCUAUTT-3’), and the target sequence of SGOL1siRNA-3# was (sense5’- CCUCAUCUUAGCCUGAAGGAUTT-3’;antisense5’-AUCCUUCAGGCUAAGAU-3’). These small-interfering RNAs (si-RNAs) were transfected using Lipofectamine 3000 (Invitrogen, Carlsbad, CA, USA), according to manufacturer’s instructions. Total RNA was extracted from HCC tissues and cell lines using TRIzol reagent (Invitrogen), and the concentration and purity of RNA were measured using a NanoDrop spectrophotometer (Thermo Fisher Science, Waltham, MA, USA). Besides, RNA reverse transcription was performed using the PrimeScript™ RT Reagent kit (TaKaRa, Shiga, Japan). Polymerase chain reaction detection systems were used with TB Green^®^ Premix Ex Taq™II kit (TakaRa). Finally, the transcript levels were normalized to those of glyceraldehyde 3-phosphate dehydrogenase (GAPDH) (an internal control gene). Primers of SGOL1 and GAPDH were (sense5′-GCCAGCGTGAACTATAAGG-3′; antisense5′-TGAAGCAACAGAAAGAGGTG-3′) and (sense5’-CCACAGTCCATGCCATCACTG-3’; antisense5 ‘ -GTCAGGTCCACCACTGACACG-3 ‘), respectively.

#### Western blotting

Total protein was isolated using radioimmunoprecipitation assay (RIPA) lysis buffer (Merck Millipore, Waltham, MA, USA) and quantified using the BCA kit (Solarbio Co., Ltd., Beijing, China) from cell lines or HCC tissues. After being subjected to sodium dodecyl sulfate-polyacrylamide gel electrophoresis (SDS-PAGE), samples were transferred onto polyvinylidene difluoride (PVDF) membranes (0.45 μm; Merck Millipore), blocked with 5% skimmed milk in Tris buffer and 0. 1% Tween-20 for 2 h, followed by incubation at 4 °Covernight with anti-SGOL1 (Proteintech, Wuhan, China), Cyclin D1 (Proteintech), Cyclin E1 (Wuhan, China), GAPDH (1:1000, Servicebio Co., Ltd., Wuhan, China), CDK7 (1:1000, Proteintech), CDK4 (1:1000, Servicebio Co., Ltd.), and P27 (1:1000, Proteintech) antibodies. Secondary antibodies were incubated for 2 h after membranes were thrice washed with TBST. Bands were displayed using the ultrasensitive ECL chemiluminescence kit (Boster Co., Ltd., Wuhan, China), and protein expression level was visualized by an imaging system (Bio-Rad Laboratories, Hercules, CA, USA).

#### Examining cell migration, invasion, and proliferation

In order to quantify cell proliferation indirectly, the cell counting kit-8 (CCK-8) and 5-Ethynyl-2’-deoxyuridine nucleoside (EDU) methods were used. Three replicate samples were used for each group, and cells were injected into 96-well plates at a density of 3*10^3^ cells/well, and each group contained 3 replicate samples. After incubation for 0, 24, 48, and 72 h, respectively, CCK-8 chromogenic solution (GlpBio Inc., Montclair, CA, USA) was added to the cells and incubated for 2 h at 37 °C. The absorbance values were recorded at 450 nm using a Quant ELISA reader (BioTek Instruments Inc., Winooski, VT, USA). EDU incorporation experiment was performed as follows: Inoculate 2*10^4^ cells per well in a 12-well plate and allow them to grow to the appropriate density. According to the Click-iT EDU-555 kit (Servicebio Co., Ltd.), the cells were incubated for 2 h after addition of 20 µM EDU storage solution and fixed in 4% paraformaldehyde. Fluorescent staining was carried out using the fluorescent dyes iF555 and DAPI, according to the instructions provided by the manufacturer. Next, a fluorescence microscope (Nikon, Tokyo, Japan) was utilized to calculate the percentage of EDU-positive cells.

The Transwell experiment and the wound healing assay were employed to examine the capacity of liver cancer cells to migrate and invade. HUH7 and HepG2 cells were digested, centrifuged, and injected into six-well plates for the wound healing experiment. These cells were delineated on 200 μL pipette dishes after they had achieved 90% fusion, thrice washed with phosphate-buffered saline (PBS), photographed using an inverted microscope, and cultured in serum-free media for 48 h before being photographed once more. Finally, the relative migration distances of each group were compared. The Transwell experiment was conducted in an 8-mm transwell chamber (NEST Co., Ltd., Wuxi, China), the cell density was adjusted to 2×10^5^ cells/mL, and 200 μL of cell suspension was inoculated into the upper chamber of serum-free medium with or without matrix gel; then, 600 μL of DMEM supplemented with 20% FBS was incorporated to the lower chamber, the cells were incubated for 36 h, twice washed with PBS, fixed in 4% paraformaldehyde for 15 min at room temperature, and stained with 0.3% crystal violet.

#### Statistical analysis

Comparisons between the two groups were statistically performed using the t-test or one-way analysis of variance (ANOVA), in which Kaplan-Meier test was used to assess patients’ survival, and the log-rank test was utilized for comparing survival-based differences. SGOL1 expression level and other clinical parameters were used to evaluate the independent prognostic significance of overall survival (OS) in HCC patients using univariate and multivariate Cox regression analyses, and the results were visualized using the “forest plot” package. The statistical analysis was performed using R 4.2.1 (R Foundation for Statistical Computing, Vienna, Austria) and SPSS 26.0 (IBM, Armonk, NY, USA) software. Statistical significance was defined as P<0.05. Continuous data were expressed as mean ± standard deviation (SD).

The following R packages were used in this study: “limma”, “edgeR”, “GSVA”, “survival”, “survminer”, “forestplot”, “ggplot2”, and “pheatmap” from Bioconductor (http://www.bioconductor.org/), and “immunedeconv” from Bioconda (https://anaconda.org/bioconda).

## Results

### Pan-cancer analysis of SGOL1 expression level

**Figure 2:**
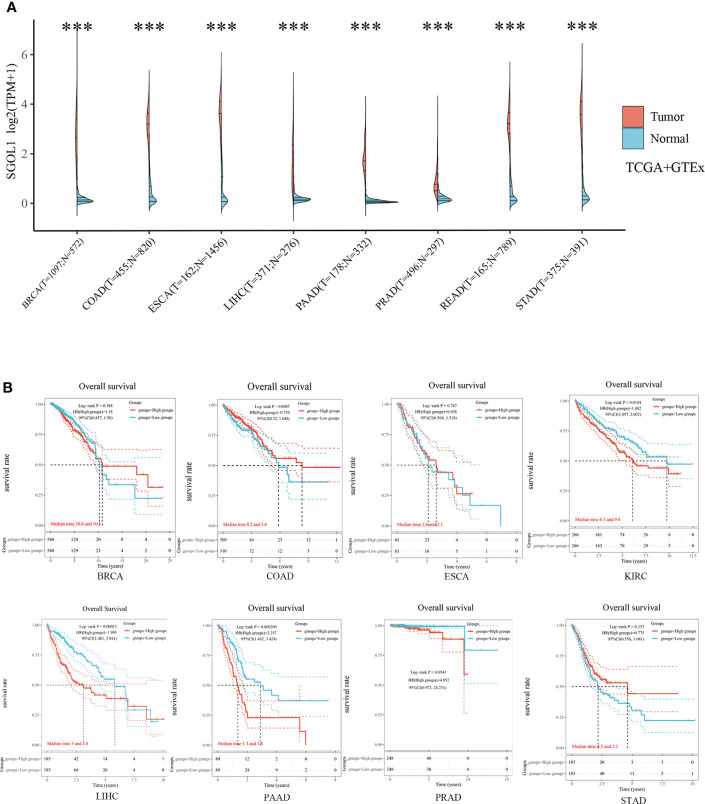
SGOL1 expression levels and prognosis in different types of human cancers. **(A)** Transcriptional expression of SGOL1 in 8 tumors with different cancer types (TCGA and GTEx). **(B)** Prognosis of SGOL1 expression in 8 different cancer types. ***p < 0.001. ‘ns’ indicates no significance.

To screen appropriate malignancies for involvenent in the study, pan-cancer analysis was performed. SGOL1 expression level in different types of cancer was determined using independent datasets from TCGA and GTEx databases *via* different sources. SGOLI expression level in human cancer tissues and their corresponding normal tissues was studied using TCGA and GTEx databases. SGOLI expression level was significantly higher in tumor (BRCA, COAD, and LIHC) tissues than that in normal tissues **(** **Figure 2A** **)**. In addition, a lower OS was found in the high expression group than that in the low expression group in KIRC, LIHC, and PAAD, indicating a poor prognosis, while no statistical difference was found with the other five malignancies, including BRCA and COAD (P > 0.05) **(** **Figure 2B** **)**.

### Relative expression level of SGOL1 in HCC tissues

**Figure 3:**
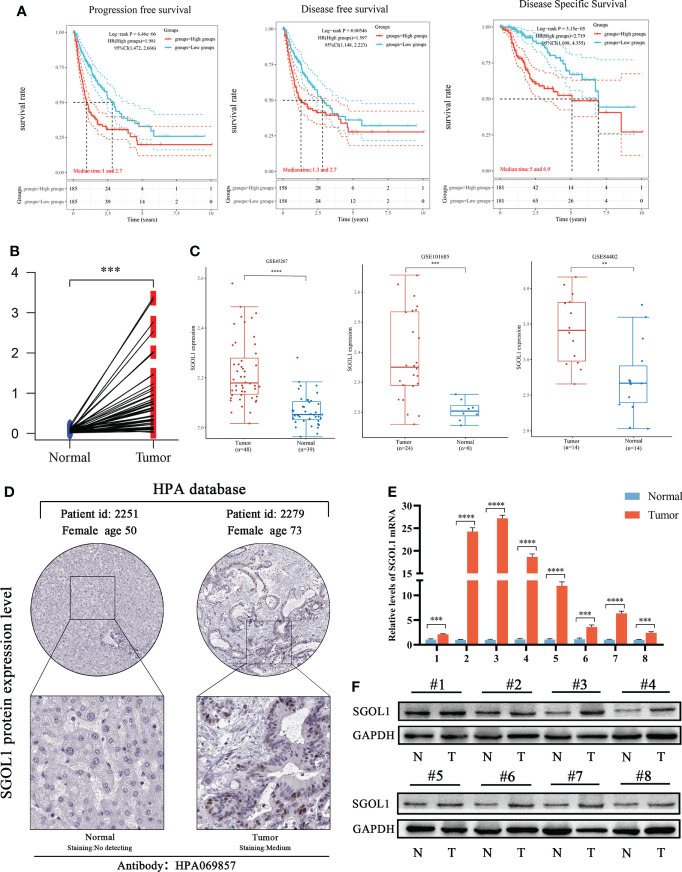
Prognosis of SGOL1 in HCC and its expression in tissues. **(A)** SGOL1 in relation to PFS, DFS, and DSS in HCC patients. **(B)** SGOL1 expression in paired HCC patients in the TCGA database. **(C)** Transcript levels of SGOL1 in GES45267, GSE101685, and GSE84402 datasets. **(D)** Immunohistochemistry of SGOL1 in HCC from the HPA database. **(E)** SGOL1 transcript levels in paired HCC clinical samples. **(F)** SGOL1 protein expression levels in paired HCC clinical samples. Statistical treatment was performed by t-test. **p < 0.01, ***p < 0.001, ****p < 0.0001.

Further analysis was performed, and it was revealed that the high SGOL1 expression level was associated with poor progression-free survival (PFS), disease-free survival (DFS), and disease-specific survival (DSS) in HCC patients **(** **Figure 3A** **)**, thus, we selected HCC for the next step of our study. As the high SGOL1 expression level in HCC tissues is associated with poor prognosis, we further validated the high SGOL1 expression level in HCC tissues using multiple databases and our clinical samples. It was found that mRNA expression level in HCC tissues was significantly higher than that in normal liver tissues in TCGA database (P<0.001) **(** **Figure 3B** **)**. This finding was also validated in three datasets of GSE45267, GSE101685, and GSE84402 in the GEO database for liver cancer and normal tissues (P<0.001) **(** **Figure 3C** **)**. Additionally, it was revealed that immunohistochemical staining of the HPA database showed positive staining for SGOL1 protein in HCC tissues **(** **Figure 3D** **)**. Finally, using qRT-PCR and Western blotting of HCC tissues and paired normal paracancerous tissues, it was confirmed that the SGOL1 expression level was significantly higher in hepatocellular carcinoma tissues than that in corresponding normal liver tissues **(** **Figures 3E, F** **)**.

### Relationship between clinicopathological characteristics of HCC patients and SGOL1 expression level

**Figure 4:**
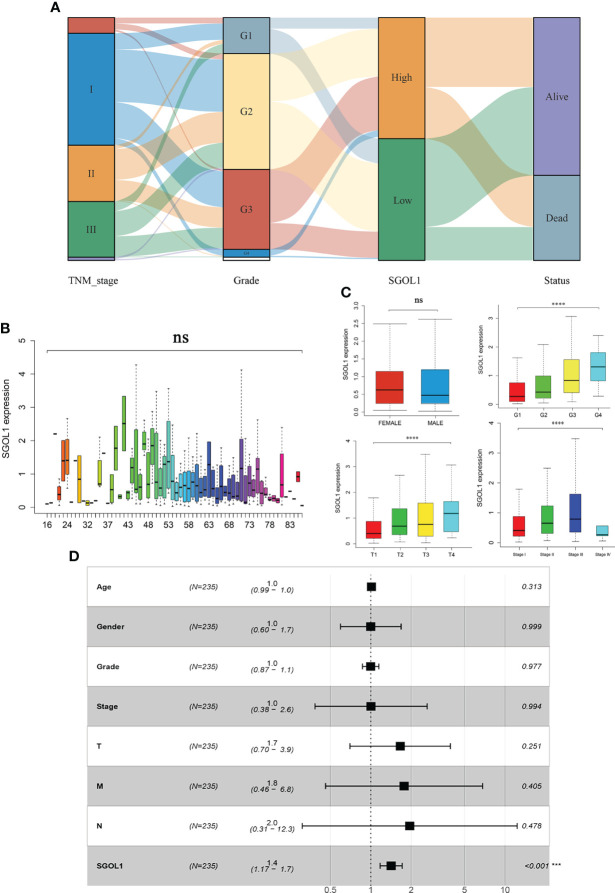
Correlation of SGOL1 expression with clinical parameters of HCC patients in TCGA database. **(A)** Relationship between SGOL1 expression and HCC stage, tumor grade, and survival status. **(B)** Relationship between SGOL1 relative expression and patient age. **(C)** Relationship between SGOL1 relative expression and gender, tumor grade, tumor size, and tumor stage. **(D)** Multi-factorial logistic regression analysis of patient prognosis. ****p < 0.0001. ‘ns’ indicates not significant.

Using clinical data from HCC patients in TCGA, which included clinical stage, tumor grade, survival status, age, gender, and pathological tumor-node-metastasis (pTNM) stage, the association between SGOL1 expression level and the clinical and pathological characteristics of HCC patients was evaluated. For stage II and III patients, tumor grades G2 and G3, a poor prognosis was mainly associated with a high SGOL1 expression level **(** **Figure 4A** **)**. Univariate analysis was carried out to explore the relationship between SGOL1 expression level and clinical indicators, and the results showed that SGOL1 expression level in HCC tissues was associated with tumor grade, tumor stage, tumor diameter, survival status, and age (P < 0.05), and there was no statistical difference with lymphatic metastasis and distant metastasis (P > 0.05) **(** **Table 1** **)**. In addition, univariate analysis of patients’ prognosis was conducted using clinical data and SGOL1 expression level. The results revealed a correlation between tumor stage, tumor grade, and tumor size in HCC patients, and the results were visualized using R software **(** **Figures 4B, C** **)**. The statistically significant clinical features were included in the multivariate logistic regression analysis, and SGOL1 expression level was found to independently influence HCC patients’ prognosis **(** **Table 2** **)**, and the results of the multivariate logistic regression analysis were visualized using a forest plot **(** **Figure 4D** **)**.

**Table 1:** Association of SGOL1 relative expression with clinical data Clinical characteristics.

Clinical characteristics		Total (N)	Odds ratio in SGOL1 expression	*P-*Value
Grade	I VS II	234	1.48 (0.79-2.86)	0.220
	I VS III	178	4.01 (2.06 - 8.03)	**<0.001**
	I VS IV	66	10.27 (2.40-71.61)	**0.004**
Stage	I VS II	262	1.77 (1.05 - 3.01)	**0.030**
	I VS III	261	2.21 (1.30 - 3.80)	**0.003**
	I VS IV	180	0.93 (0.12 - 5.80)	0.945
Tumor diameter	T1 vs T2	275	1.99 (1.20 - 3.32)	**0.007**
	T1 vs T3	261	2.21 (1.30 - 3.82)	**0.003**
Status (free or with)		371	1.77 (1.05 - 3.01)	**0.030**
Age (continuous)		371	0.98 (0.96-0.99)	**0.044**
Gender (female VS male)		371	0.72 (0.46 - 1.11)	0.140
Distant metastasis		270	0.32 (0.01 - 2.60)	0.337
Lymph nodes		256	1.00 (0.12 - 8.43)	1.000

Bold values indicate significant p-values.

**Table 2:** Logistic regression analysis of survival prognosis against clinical data and SGOL1.

Parameter	Univariate analysis			Multivariate analysis		
	HR	95% CI	*P-*Value	HR	95% CI	*P-*Value
Age	1.00	0.98 - 1.02	0.591			
Gender	0.78	0.48 - 1.25	0.301			
Grade	0.97	0.85 - 1.11	0.663			
Stage	1.86	1.46 - 2.38	**<0.001**	1.22	0.52 - 2.84	0.636
T	1.80	1.43 - 2.27	**<0.001**	1.38	0.64 - 2.98	0.410
M	3.84	1.20-12.28	**0.022**	1.71	0.46 - 6.29	0.417
N	2.02	0.49 - 8.27	0.327			
SGOL1	1.49	1.25 - 1.77	**<0.001**	1.40	1.16 - 1.69	**0.0004**

Bold values indicate significant p-values.

### Enrichment analysis of SGOL1 in HCC

**Figure 5:**
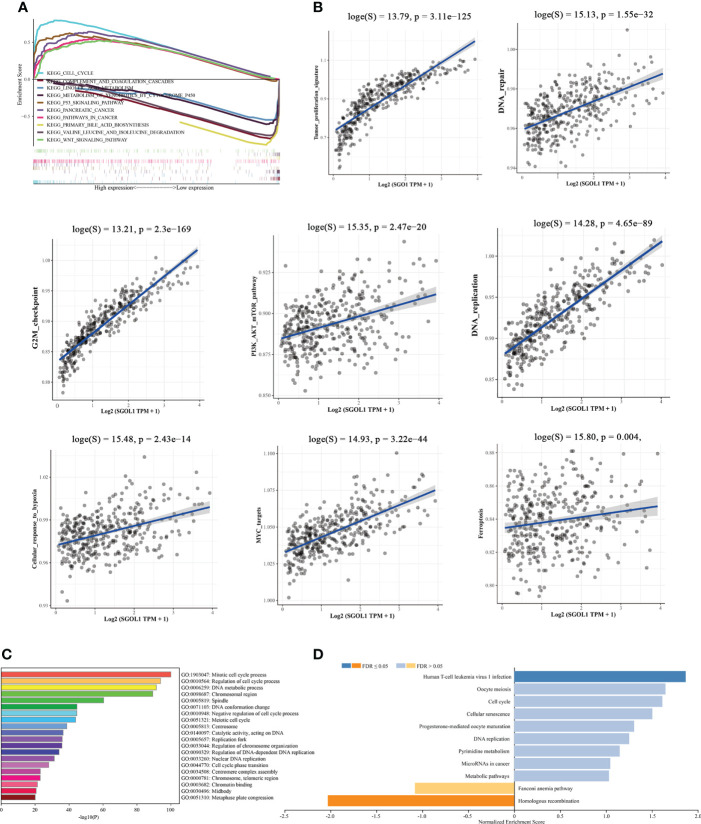
Functional and mechanistic enrichment analysis of SGOL1 in HCC. **(A)** GESA enrichment map of SGOL1 integration in HCC, including 5 up-regulated pathways and 5 down-regulated pathways; **(B)** Relationship between SGOL1 and tumor and cycle pathways in hepatocytes **(C)** GO enrichment map of GOL1 integration in HCC. **(D)** Pathways in which SGOL1 is mainly involved.

To further elucidate the possible mechanisms of SGOL1 in the progression of HCC, the Kyoto Encyclopedia of Genes and Genomes (KEGG) pathway analysis revealed several major pathways, including five upregulated pathways and five downregulated pathways. The upregulated pathways mainly contained cell cycle, P53 signaling pathway, pancreatic cancer, tumor pathway, Wnt signaling pathway, and the downregulated pathways mainly included linoleic acid metabolic pathway, complement, and coagulation cascade, cytochrome P450 on exogenous bio metabolism, degradation of valine, leucine, and isoleucine, and primary bile acid synthesis **(** **Figure 5A** **)**. The results of the KEGG pathway analysis indicated that SGOL1 expression level was mainly associated with cell cycle, DNA replication, and tumor pathways in HCC. Therefore, we further analyzed the tumor-related pathways and found that SGOL1 expression level was associated with tumor proliferation characteristics, DNA repair, G2M checkpoint, PI3K-AKT-mTOR signaling pathway, DNA replication, cellular response to hypoxia, and MYC target genes **(** **Figure 5B** **)**. The GO enrichment analysis and normalized data (FDR < 0.05) also supported the involvement of SGOL1 in mitosis, DNA replication, cell cycle processes, human T-cell leukemia virus type 1 infection, etc. **(** **Figures 5C, D** **)**.

### Relationship between immune cells and SGOL1 expression level in HCC tissues

**Figure 6:**
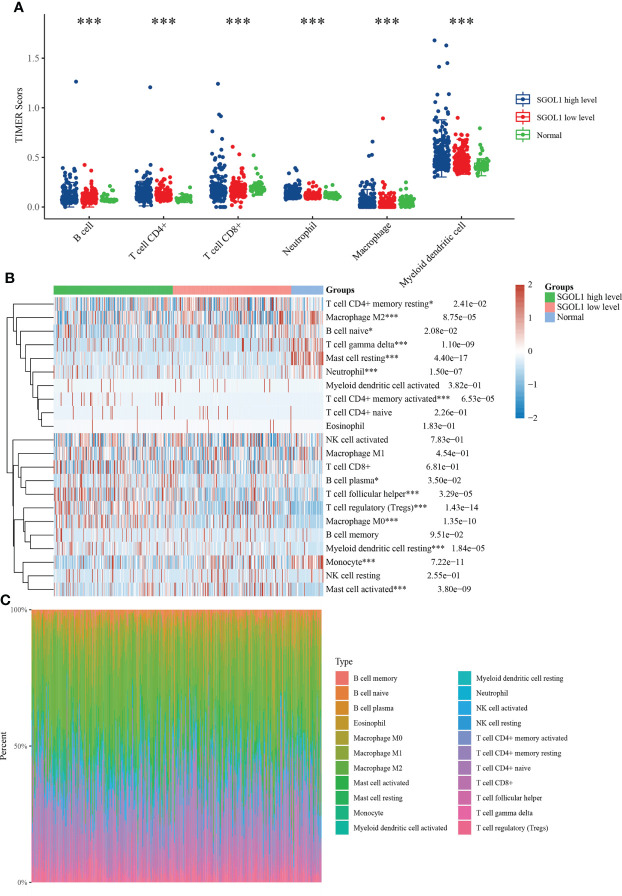
Relationship between SGOL1 expression and tumor immune cell infiltration in HCC. **(A)** Distribution of SGOL1 versus immune cells in HCC versus normal tissues based on the TIMER algorithm. **(B)** Evaluation of the distribution of SGOL1 versus T cells in HCC versus normal tissues based on the CIBERSORT algorithm. **(C)** Percentage abundance of SGOL1 versus tumor-infiltrating immune cells in HCC versus normal tissues for each sample. Statistical treatment: two samples significant by Wilcox test, three and more samples significant by Kruskal-Wallis test, *p < 0.05, ***p < 0.001.

**Figure 7:**
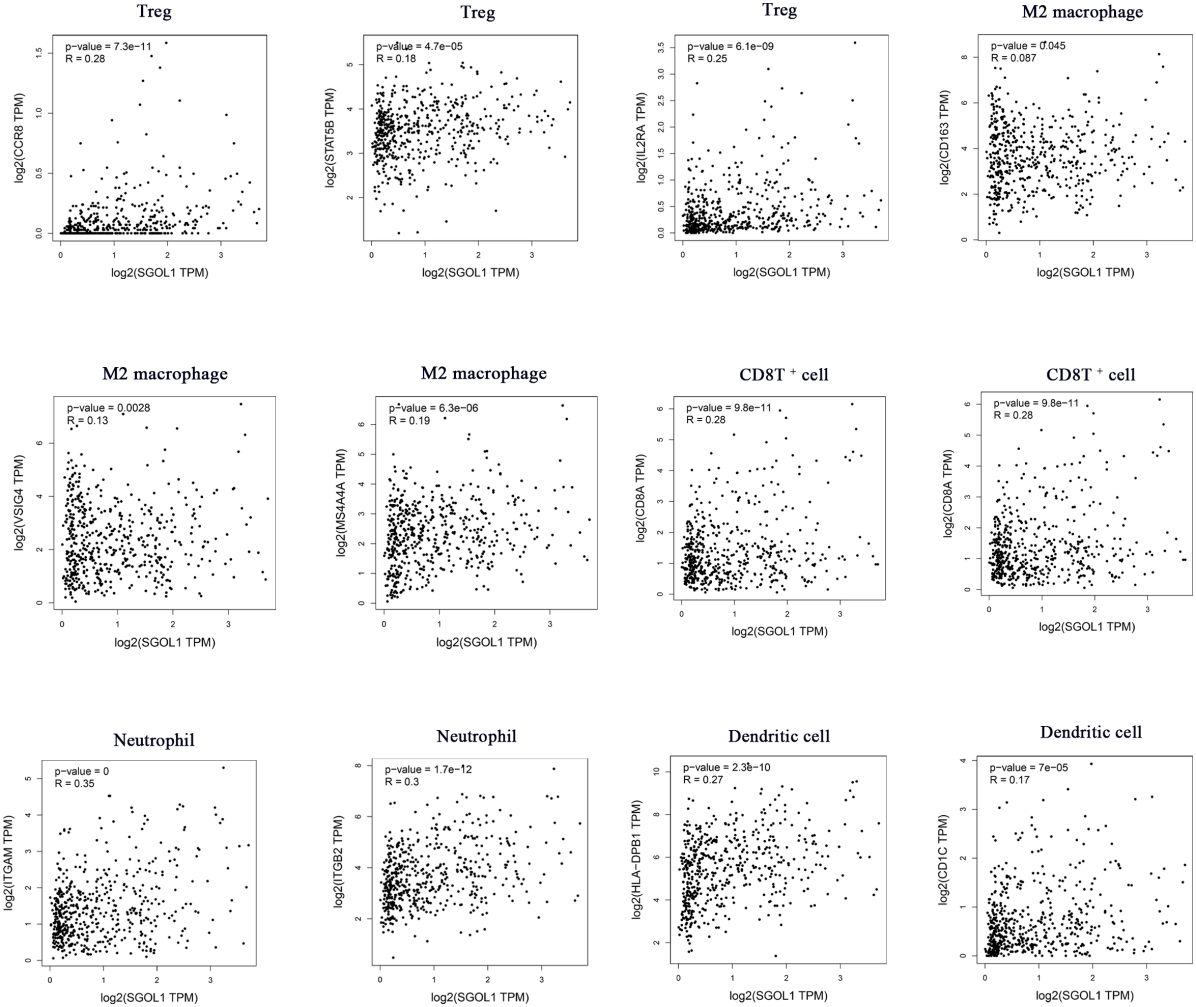
Association of SGOL1 with Treg, M2 macrophages, CD8T+ cells, neutrophils, and dendritic cell markers in HCC.

Innate and adaptive immune system cells play a major role in regulating the growth of cancer (19). The association between SGOL1 expression level and immune infiltrating cells in HCC has still remained elusive. Therefore, R software was utilized to analyze the relationship between SGOL1 expression level and immune cell infiltration, as well as the proportional distribution of immune cells in normal liver tissue adjacent to cancer tissue. In accordance with the findings **(** **Figure 6A** **)**, HCC tissues with a high SGOL1 expression level had a substantially higher number of B cells, CD4^+^ T cells, CD8^+^ T cells, neutrophils, macrophages, and myeloid dendritic cells (P<0.001). Further assessment of T cells using the CIBERSORT algorithm showed that M2 macrophages, δT cells, memory-activated CD4^+^ T cells, regulatory T cells (Tregs), B plasma cells, T follicular helper cells, M0 macrophages, myeloid dendritic cells, monocytes, and activated mast cells were associated with SGOL1 expression level **(** **Figures 6B, C** **)**. With GEPIA2 analysis of SGOL1 expression level with biomarkers of Treg cells (STAT5B, CCR8, and IL2RA), CD8^+^ T cells (CD8A and CD8B), M2 macrophages (CD163, VSIG4, and MS4A4A), neutrophils (ITGAM and CCR7), and dendritic cells (DCs; HLA-DPB1, CD1C) in HCC tissues, a significantly positive correlation was found between SGOL1 expression level and the above-mentioned biomarkers of immune infiltrating cells **(** **Figure 7** **)**.

### Relationship between immune checkpoints and SGOL1 expression level in HCC tissues

**Figure 8:**
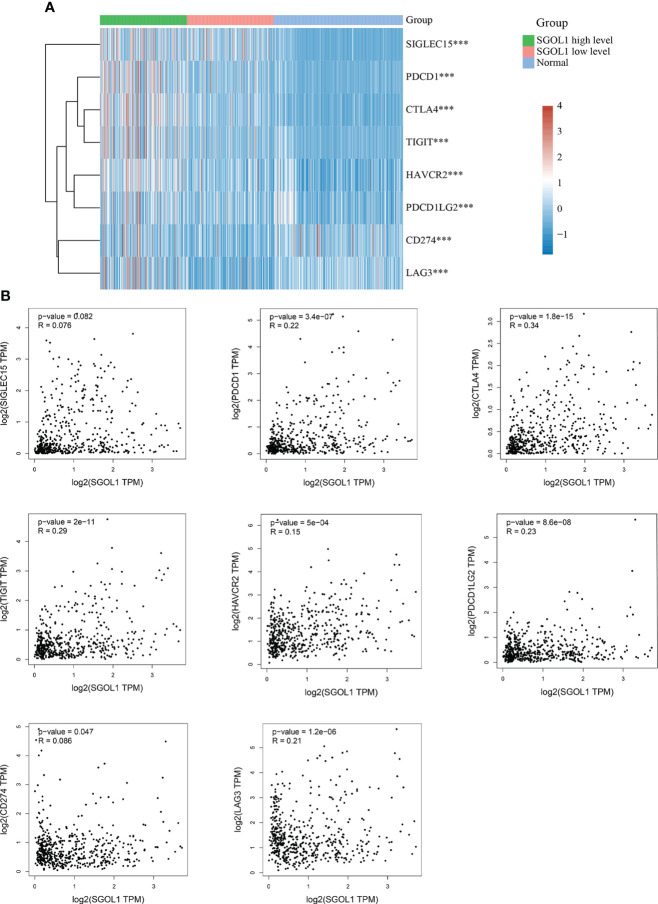
Relationship of SGOL1 to immune checkpoints in HCC; **(A)** SGOL1 expression in tumor and normal tissues in association with immune checkpoint-related gene expression. **(B)** Association of SGOL1 with immune checkpoint-associated genes in liver cancer tissues. ***p < 0.001.

Studies have shown that tumor immune escape is one of the important causes of cancer development, which limits the activation of T cells by binding to immune checkpoints (20). We, in the present study, determined SGOL1 expression level in HCC and adjacent normal tissues in association with SIGLEC15, TIGIT, CD274, HAVCR2, PDCD1, CTLA4, LAG3, and PDCD1LG2 in light of the possible oncogenic involvement of SGOL1 in HCC. The findings demonstrated that SGOL1 expression level was correlated with each of these immune checkpoints (P < 0.001) **(** **Figure 8A** **)**. Additionally, the GEPIA2 website was utilized to further examine the relationship between the expression levels of the immune checkpoints and SGOL1 expression level, and a positive correlation was identified between the transcript levels of the immune checkpoints **(** **Figure 8B** **)**. These results indicated the relationship between tumor immunity and SGOL1 expression level in HCC tissues.

### Silencing of SGOL1 gene inhibited tumorigenicity of HCC cells *ex vivo*

**Figure 9:**
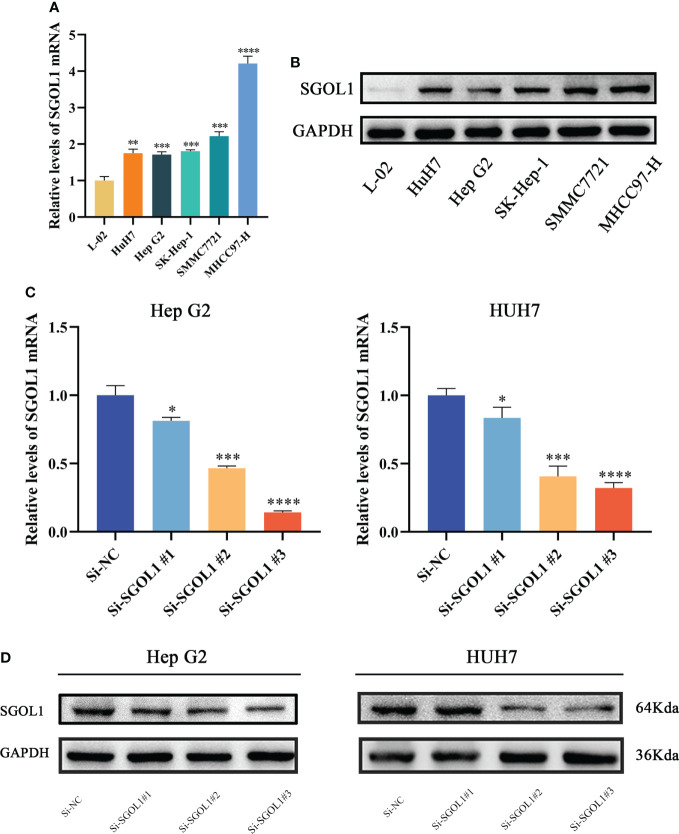
SGOL1 expression in hepatocytes and construction of silent SGOL1 cells. **(A)** Transcript levels of SGOL1 in LO2 and hepatocellular carcinoma cells. **(B)** Protein expression levels of SGOL1 in LO2 and hepatocellular carcinoma cells. **(C)** Validation of interfering RNA efficiency transcript levels. **(D)** Validation of protein levels of interfering RNA efficiency. *p < 0.05, **p < 0.01, ***p < 0.001, ****p < 0.0001.

We first assessed the results using qRT-PCR of HCC cell lines and L02 in healthy human hepatocytes to confirm SGOL1 expression level in HCC cells. The findings demonstrated that SGOL1 expression level was significantly upregulated in HCC cells compared with that in L02 cells **(** **Figure 9A** **)**, and Western blotting confirmed these findings **(** **Figure 9B** **)**. We selected two cell lines, HepG2 and HUH7, to investigate the effect of SGOL1 expression level on the proliferation, migration, and invasion of HCC cells. HepG2 and HUH7 cells were transfected with si-RNA, and the results were verified by qRT-PCR. The findings revealed that SGOL1 expression level was reduced in three experimental groups compared with the control si-NC, in which si-SGOL1#3 showed the most significant reduction **(** **Figure 9C** **),** and Western blotting further corroborated the findings of gene silencing **(** **Figure 9D** **)**; thus, si-SGOL1#3 was utilized for further research, and the results indicated that the gene silencing was effective.

**Figure 10:**
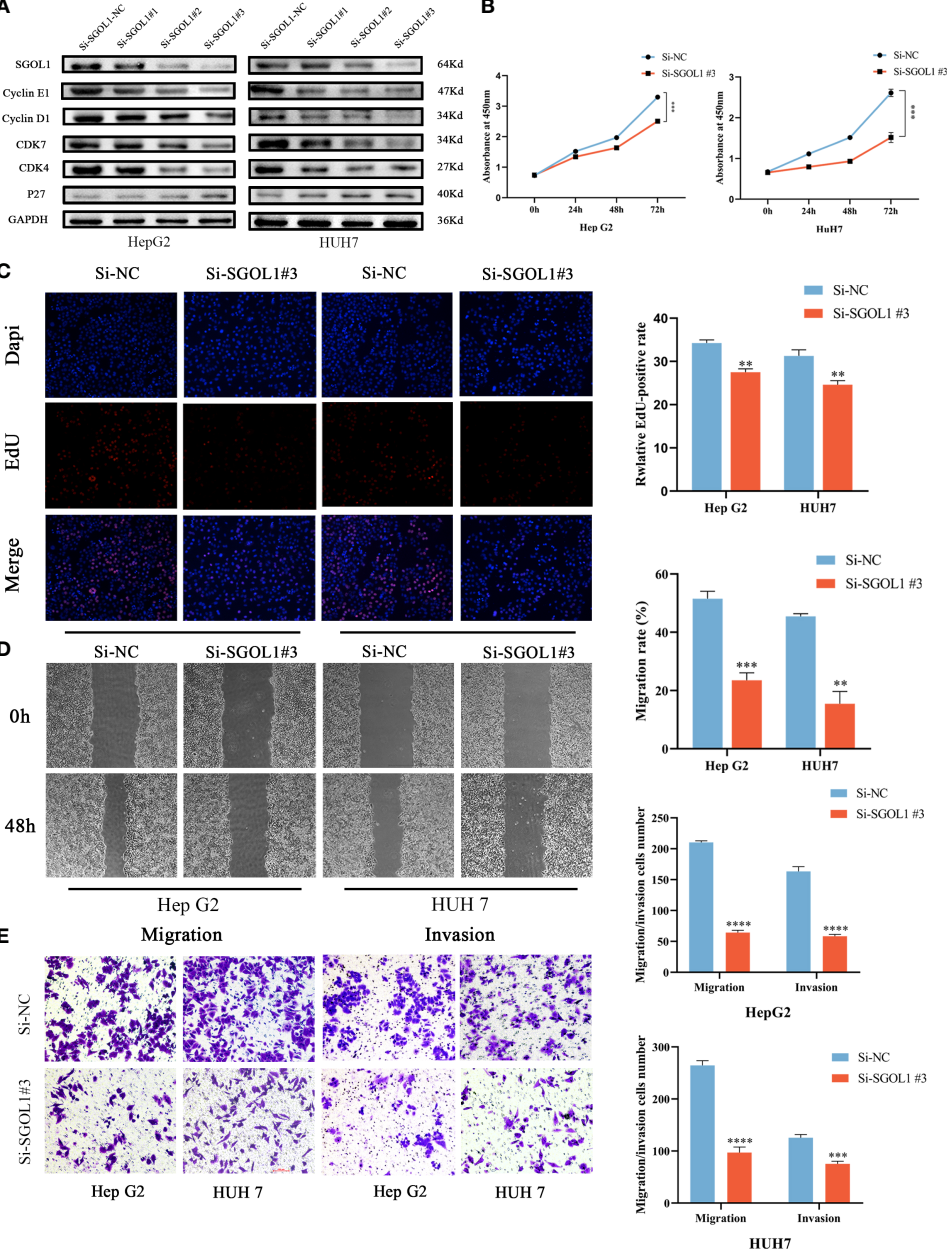
Effect of silencing SGOL1 on proliferation and cell motility of hepatocellular carcinoma cells. **(A)** Western blotting to detect the expression of cell cycle markers in hepatocellular carcinoma cells after SGOL1 gene silencing. **(B)** Changes in cell proliferation at 0, 24, 48 and 72 hours after SGOL1 gene silencing in cells were examined using the CCK-8 assay. **(C)** EDU doping assay inhibited the proliferation of hepatocellular carcinoma cells after silencing the SGOL1 gene. **(D)** Wound healing assay detects inhibition of migration ability of hepatocellular carcinoma cells after silencing of SGOL1 gene. **(E)** Transwell assay detects diminished migration and invasion ability of hepatocellular carcinoma cells after silencing the SGOL1 gene. Data generated from three independent experiments, expressed using mean ± SD, **p < 0.01, ***p < 0.001, ****p < 0.0001.

We examined the effect of silencing of SGOL1 gene on the expression levels of the Cyclin E1, Cyclin D1, CDK7, CDK4, and P27 in HepG2 and HUH7 cells using Western blotting. According to the findings, when SGOL1 expression level was downregulated in HepG2 and HUH7 cells, the expression levels of Cyclin E1, Cyclin D1, CDK7, and CDK4 were downregulated, while P27 expression level was upregulated **(** **Figure 10A** **)**. Meanwhile, the CCK-8 assay revealed that silencing of SGOL1 gene inhibited the proliferation of HepG2 and HUH7 cells **(** **Figure 10B** **)**, which was further validated by the EDU assay **(** **Figure 10C** **)**. We employed wound healing and Transwell assays to detect migration and invasion capabilities of HepG2 and HUH7 cells in association with SGOL1 expression level. The results showed that, compared with the control, silencing of SGOL1 significantly reduced the motility of HepG2 and HUH7 cells **(** **Figure 10D** **)**. Transwell assay further confirmed the inhibition of motility of HCC cells by silencing of SGOL1 **(** **Figures 10E, F** **)**. Taken together, the proliferation, migration, and invasion capabilities of HepG2 and HUH7 cells significantly decreased when the SGOL1 gene was silenced.

## Discussion

The incidence of HCC is high, its prognosis is poor, and it is mainly diagnosed at advanced stages. Therefore, it is extremely important to understand molecular mechanisms of HCC and to find HCC-associated prognostic biomarkers.

A growing body of evidence demonstrated that SGOL1 is essential for the development and progression of several human malignancies, including HCC (21). However, few studies have concentrated on SGOL1 expression level in HCC cells and tissues, and further research is therefore required. SGOL1 has been found to contribute to the accurate division of stabilized chromosomes and to coordinate chromosomal segregation in mitosis and meiosis (22), a crucial mechanism for the error-free segregation of chromosomes. Essential processes in cancer, progression, and invasion include aberrant DNA replication and uncontrolled cell cycle. Iwaizumi et al. (23) induced CIN in colorectal cancer cells through SGOL1 downregulation. We, in the present study, performed the pan-cancer analysis of SGOL1 expression level using data from TCGA and GTEx databases, and it was revealed that SGOL1 expression level was upregulated in the majority of the types of cancer and significantly decreased in normal tissues compared with that in HCC tissues. The GEO database confirmed the high SGOL1 expression level in HCC tissues. It was revealed that SGOL1 was highly expressed in almost all tumors, while with different degrees. This may be related to the different stages of cell growth of SGOL1 or the existence of different molecular pathways in different tumors. Hence, further research is required to clarify the role of SGOL1 expression level in HCC.

The survival study showed that patients with the high SGOL1 expression level in HCC tissues had poorer OS, PFS, DFS, and DSS. In addition, after collating clinical data from patients in TCGA, it was found that SGOL1 expression level was associated with proliferation of tumor cells and was an independent predictor of poor prognosis in HCC patients, suggesting that SGOL1 is an oncogene in HCC and its overexpression promotes tumor progression.

According to previous studies, the liver is a sophisticated immunological organ that contains a variety of immune cells, including Kupffer cells (KCs), natural killer (NK) cells, DCs, etc. (2). Malignant tumor formation, progression, and metastasis are all intimately correlated with the tumor microenvironment (TME), and HCC is no exception (24, 25). The majority of HCC cases are accompanied with persistent inflammation and fibrosis confirmed by a variety of etiologies, including viral hepatitis, excessive alcohol use, and non-alcoholic liver disease (26), massive groups of immune cells eliminate the pathogens, endotoxins, and chemokine-mediated inflammatory responses that invade the liver during prolonged chronic inflammation (27). These immune cells infiltrate inside the liver for a long time, causing additional immune-mediated damage that alters hepatocyte proliferation and stimulates the development of hepatocarcinogenesis, as well as causing DNA damage, genetic instability, and proliferation-related replicative stress (28, 29).

In HCC, the infiltration of several factors and immune cells plays a negative or positive regulatory role in its progression and development, affecting tumor proliferation (30). Based on our findings on the biological transactivity of the complex interactions between immune cells and HCC, SGOL1 is involved in the control of the T-cell leukemia virus type 1 oncoprotein, which is a direct target of the human T-cell leukemia virus type 1 oncoprotein regulating mitotic arrest defect-like 1 (MAD1), and it is closely associated with CIN, thereby participating in tumor progression or cellular senescence (31). To date, no correlation between SGOLI expression level and tumor immunity in HCC has been suggested. Therefore, we assessed the relationship between SGOL1 expression level in HCC tissues and immune cell infiltration (32), and it was shown that SGOL1 expression level in HCC tissue was associated with the number of B cells, macrophages, neutrophils, CD4^+^ T cells, CD8^+^ T cells, and DCs. Secondly, the association of SGOL1 expression level with the expression levels of these immune cells-related markers (e.g., Tregs, M2 macrophages, CD8^+^ T cells, neutrophils, and dendritic cells) was evaluated by GEPIA, and it was revealed that SGOL1 expression level was positively correlated with biomarkers of immune cells in HCC. Tumor-associated macrophage (TAM) can promote HCC development and progression, which is characterized by an M2 polarized phenotype that facilitates invasion and migration of HCC cells *in vitro*, and it plays a significant role in TME (33). Additionally, Tregs and M2 macrophages support immune escape, worsen the prognosis of HCC patients, and aid in the immune system’s protection against CD8^+^ T cell-mediated anti-tumor immunological type responses (34). According to the results, we hypothesized that SGOL1 could govern the immune infiltration of HCC cells and further affect the prognosis of HCC *via* its relationship with the expression levels of these immune cells-related markers.

Recent studies have shown that immunological checkpoints play a role in the development of diverse types of cancer, primarily by modifying immune evasion and inhibiting antitumor immune responses in solid tumors (35). At present, the most frequently identified and utilized immune checkpoints in research and clinical applications are cytotoxic T-lymphocyte-associated antigen 4 (CTLA-4), programmed cell death protein-1 (PD-1), and programmed cell death ligand 1 (PD-L1), which are all involved in immunity primarily through regulation of T cells. Inhibition of their expression levels may improve patient prognosis to some extent (36, 37). We found that SGOL1 expression level varied between HCC and normal tissues, and then, analyzed the association between SGOL1 expression level and expression levels of immune checkpoints using GEPIA2, which revealed a positive association for all genes with the exception of SIGLEC15. We hypothesized that SGOL1 expression level could be correlated with the expression levels of immune checkpoints in HCC tumors to achieve immune evasion by suppressing T cell function, further promoting HCC progression, which is a complex process, and the exact promotion mechanism still needs to be clarified. These results may partially explain the oncogenic function of SGOL1 in promoting immune evasion in HCC.

To further assess the effect of SGOL1 expression level on proliferation and invasion of HCC cells, we first validated the high SGOL1 mRNA and protein levels in HCC tissues and cell lines derived from patients. Next, we established a model of SGOL1 gene silencing in HepG2 and HUH7 cells and found, *via in vitro* tests, that silencing of SGOL1 gene inhibited the proliferation, migration, and invasion of HCC cells. SGOL1 plays a crucial regulatory role in the cell cycle and mitosis (38). As a result, we assessed how silencing of SGOL1 gene could affect cell cycle proteins. The present study demonstrated that when SGOL1 expression level was suppressed, the expression levels of Cyclin E1, Cyclin D1, CDK7, and CDK4 in HepG2 and HUH7 cells were significantly lower than those in the control group, while P27 expression level was significantly higher, suggesting that cell proliferation was constrained. In the meantime, CCK8 and EDU experiments further demonstrated that silencing of SGOL1 gene hindered the proliferation capacity of HCC cells. Additionally, it was revealed that silencing of SGOL1 gene decreased the capability of HepG2 and HUH7 cells to invade and heal wounds. Therefore, we demonstrated by the aforementioned results that silencing of SGOL1 gene increased proliferation, invasion, and migration of HCC cells *in vitro*.

Although the overexpression of SGOL1 in HCC and its correlation with a poor patient prognosis were assessed in the present study, and several mechanisms of its growth in HCC were briefly discussed, there are still some limitations. First, we used a public database of tumor samples, which may contain error-prone information. Although we utilized our clinical samples to determine transcriptional and translational levels for SGOL1, no additional research was conducted in conjunction with our immunohistochemistry and prognosis analysis. The investigation of the mechanism underlying the association of SGOL1 expression level and HCC progression and its relationship with immune infiltration is now limited to bioinformatics research, with no experimental validation. In the meantime, we only conducted *in vitro* experiments, lacking *in vivo* experiments, and further in-depth studies should be conducted in the future.

## Conclusions

In conclusion, the expression level and prognostic role of SGOL1 in several malignancies were assessed, including HCC, and the mechanisms of SGOL1’s role in HCC development and related carcinogenesis, as well as immunologically relevant mechanisms were discussed. Moreover, it was found that silencing of SGOL1 gene could inhibit the proliferation, invasion, and migration of HCC cells, suggesting that SGOL1 may be a novel target for the early detection and treatment of HCC patients, providing new insights into improve HCC patients’ prognosis.

## Data availability statement

The original contributions presented in the study are included in the article/supplementary material. Further inquiries can be directed to the corresponding author.

## Ethics statement

The studies involving human participants were reviewed and approved by Ethics Committee of Guizhou Medical University. The patients/participants provided their written informed consent to participate in this study.

## Author contributions

The study was designed by XF and YP, and XF wrote the manuscript. YP supervised the study. XF, SL and PL performed the bioinformatics analysis. CZ and XW revised the manuscript. JC and JH were involved in data collection. All authors contributed to the article and approved the submitted version.

## Funding

This study were financially supported by the National Natural Science Foundation of China (NSFC No. 81960431) and the National Natural Science Foundation of China (NSFC No. 81960535).

## Conflict of interest

The authors declare that the research was conducted in the absence of any commercial or financial relationships that could be construed as a potential conflict of interest.

## Publisher’s note

All claims expressed in this article are solely those of the authors and do not necessarily represent those of their affiliated organizations, or those of the publisher, the editors and the reviewers. Any product that may be evaluated in this article, or claim that may be made by its manufacturer, is not guaranteed or endorsed by the publisher.
